# Magnetic Resonance Imaging-Guided Treatment of Equine Distal Interphalangeal Joint Collateral Ligaments: 2009–2014

**DOI:** 10.3389/fvets.2016.00073

**Published:** 2016-09-05

**Authors:** Nathaniel A. White, Jennifer G. Barrett

**Affiliations:** ^1^Marion duPont Scott Equine Medical Center, Virginia-Maryland College of Veterinary Medicine, Virginia Tech, Leesburg, VA, USA

**Keywords:** magnetic resonance imaging, lameness-equine, desmitis, regenerative medicine

## Abstract

**Objectives:**

To determine the outcome of treating distal interphalangeal joint collateral ligament (DIJCL) desmopathy using magnetic resonance imaging (MRI)-guided ligament injection.

**Methods:**

Medical records of 13 adult horses diagnosed with DIJCL desmopathy using low-field MRI and treated by MRI-guided ligament injection of mesenchymal stem cells and/or platelet-rich plasma (PRP) were reviewed. Information collected included signalment, MRI diagnosis, treatment type, time to resolution of lameness, and level of exercise after treatment.

**Results:**

Collateral ligament inflammation was diagnosed as a cause of lameness in 13 horses. MRI was used to guide the injection of the injured DIJCL. All lameness attributed to DIJCL desmopathy resolved with the resulting level of performance at expected (10) or less than expected (3).

**Conclusion and clinical relevance:**

Injection of the DIJCL can be safely completed in horses standing in a low-field magnet guided by MRI as previously demonstrated in cadaver specimens. The positive response in all horses suggests that administration of stem cells or PRP along with rest and appropriate shoeing may be a safe and useful treatment for DIJCL desmopathy.

## Introduction

Treatment of tendon and ligament injuries by injection with either mesenchymal stem cells (MSC) or platelet-rich plasma (PRP) is reported to improve healing and eventual outcome (1, 2). These techniques have been used in tendons and ligaments of the metacarpus and metatarsus, where ultrasound can be used to direct the injection (1). Resolution of tendon and ligament fiber disruption is often monitored using sequential ultrasonography, which indicates improved fiber structure following natural disease and experimentally created lesions (3, 4).

Magnetic resonance imaging (MRI) has recently identified and characterized tendon and ligament injuries in the horse’s foot as a cause of lameness (5–9). Characteristic MRI findings of desmopathy in the distal interphalangeal joint collateral ligament (DIJCL) include ligament enlargement, changes in border definition, and increased signal intensity within the ligament (10). These changes correspond to degenerative changes observed histologically, including collagen degeneration, fissure formation, and fibrocartilaginous metaplasia, and are often accompanied with osseous change in the ligament insertion (10, 11).

Treatments for tendon and ligament injury in the foot include rest, anti-inflammatory treatment, including intra-synovial injection of corticosteroids, hyaluronic acid, interleukin receptor antagonist,^1^ supportive shoeing, and shockwave treatment (12, 13). Newer approaches to treat tendons and ligaments using regenerative medicine therapies include PRP and MSC therapy (2, 14, 15). PRP injection improved the ultrasonographic appearance and organization of the linear fiber pattern in a surgical model of superficial digital flexor tendon injury (16). Bone marrow-derived MSC treatment improved histological signs of healing and structural organization in a collagenase model of superficial digital flexor tendon injury (4). A large retrospective study looking at MSC therapy for superficial digital flexor tendon injury in National Hunt racehorses in the United Kingdom reported an 82% long-term success rate (2).

Application of regenerative therapies to DIJCL lesions is limited due to limited access in the foot. Although ultrasonography, radiography, or computed tomography may assist in localizing these therapies to lesions within the foot, each technique has distinct disadvantages. Ultrasonography gives a limited view of the DIJCL, and lesions diagnosed by MRI have been seen at the distal insertion, where the ligament cannot be seen ultrasonographically (10, 11). Radiography can assist placement of a needle in the expected area of the lesion; however, the ligament and lesion are not visible, making this a blind technique relying on anatomic understanding rather than direct visualization (17). Computed tomography with contrast enhancement can be used to specifically locate the site for injection; however, this must be performed under general anesthesia and does not offer detection of new fluid at the injection site postinjection (18).

Magnetic resonance imaging-guided techniques used successfully in human medicine for biopsies and treatment of tumors (19, 20). Recently, a technique of using MRI to guide injection of the DIJCL was developed and validated in horse feet from cadavers (21). We hypothesized that naturally occurring lesions in the DIJCL within the hoof can be accurately injected *via* MRI guidance in the standing horse. Additionally, we hypothesized that the injection technique would not compromise healing of the DIJCL healing.

## Materials and Methods

### Animals

Client-owned horses with unilateral lameness localized to the hoof *via* diagnostic nerve blocks were admitted to the Marion duPont Scott Equine Medical Center at the Virginia-Maryland College of Veterinary Medicine. All horses included in the study had moderate-to-severe uniaxial DIJCL desmopathy. When affected ligaments had evidence of severe injury or previous treatments such as rest, anti-inflammatory treatments, and corrective shoeing were not successful, ligament injection using MRI guidance was offered as a treatment. Either MCS combined with PRP or PRP alone was selected for injection based on the clinician’s preference and cost to the client.

### Diagnostic Magnetic Resonance Imaging

The diagnosis of DIJCL lesions associated with foot lameness was made using a 27-T magnet^2^ with horses in a standing position using a hoof coil (see text footnote 2). The MRI examination of the feet was completed, as previously described, using proton density-weighted spin echo, T1-weighed gradient echo sequence (T1W-GE), T2-weighted fast spin echo, and short-tau inversion-recovery fast spin echo sequence (STIR FSE) sequences (22). Proton density-weighted spin echo scans were completed in a transverse plane; T1W-GE scans in transverse, frontal, and sagittal planes; T2-weighted fast spin echo scans in dorsal and transverse planes and STIR FSE scans in transverse, dorsal, and sagittal planes.

### Preparation of Platelet Rich Plasma

Blood (450 ml) was aseptically collected in citrate–phosphate–dextrose–adenine anticoagulant from each patient for PRP processing. Blood was then processed to produce 12 ml of PRP (5- to 7-fold increase in platelets with 0.02- to 0.05-fold white blood cells) *via* centrifugation.

### Preparation of Mesenchymal Stem Cells

Bone marrow aspirate (*n* = 7) was collected aseptically from the patient’s left tuber coxa or mid sternum, as previously described (23). In brief, heparinized bone marrow was collected from the patient, the aspirate was centrifuged and cultured in low-glucose Dulbecco’s Modified Eagle Medium supplemented with 10% fetal bovine serum, 300 μg of l-glutamine/ml, 100 U of sodium penicillin, and 100 μg of streptomycin sulfate/ml at 37°C in a 5% carbon dioxide atmosphere with 90% humidity for media supplementation every 48 h. Cells were passaged at 80% confluence, and passage 1 or 2 was used for injection. Once adequate cells were prepared, the injection was scheduled, and on the day of injection, cells were trypsinized, washed with phosphate buffer solution four times, and suspended in autologous PRP for injection (5 million cells/ml).

### MRI-Guided Injection

**Figure 1:**
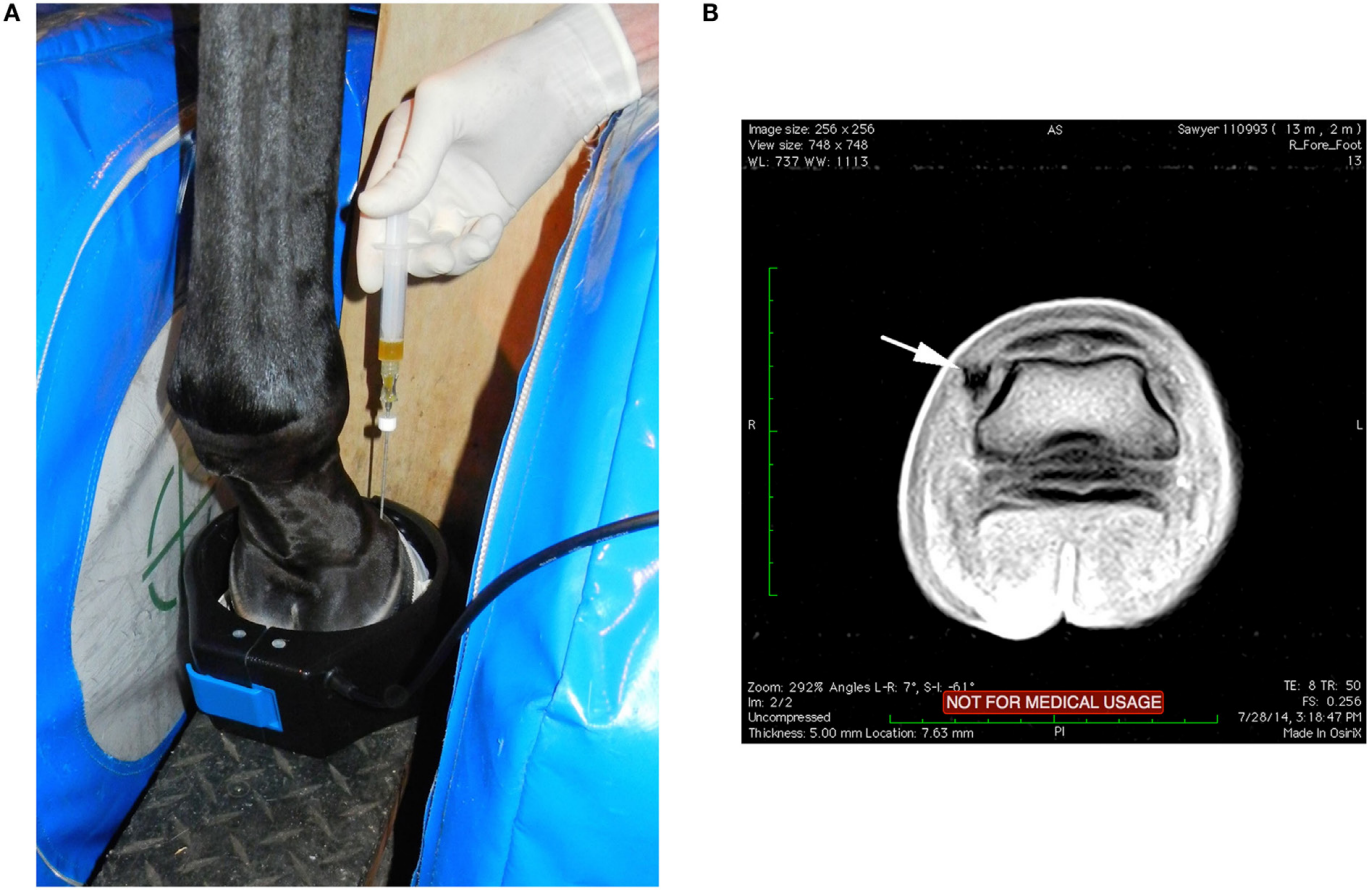
**(A)** With the horse’s left foot in the magnet the needle was placed using T1W-GE sequences to direct the needle into the medial DIJCL. **(B)** T1-weighted gradient echo sequence in this horse’s foot (medial is to the left) with focal hypointense signal (arrow) from the titanium needle placement in the DIJCL.

Local anesthesia of the digital nerves was completed at the level of the proximal sesamoid bones. Sedation for the procedure was predominately titration of intravenous detomidine and butorphanol used during routine MRI examinations of the feet and as needed to prevent movement of the horse. Aseptic preparation and wrapping of the injection site were completed prior to putting the limb in the magnet. T1W-GE and STIR FSE sequences were completed prior to injection. Injection of each DIJCL was completed using a 16-gauge intravenous catheter over a needle (3 cases) or a 16-gauge non-ferromagnetic (titanium) needle^3^ (10 cases) designed for biopsy guided by MRI. The needle placement utilized the previously described technique (21). Briefly, needle insertion was at a point between the common digital extensor tendon and the dorsal edge of the collateral cartilage and directed perpendicular to the solar surface (Figure 1). Injections were well tolerated by the horses, and there was no evidence that the horse had any sensation during the injection. Firm resistance was present as the needle penetrated the ligament. The needle was inserted until it was in contact with the coffin bone. There was resistance to injection until the needle was withdrawn a few millimeters. T1-weighted gradient echo sequences were repeated as the needle was advanced identifying the position of the needle and allowing for any needed correction of its position. In the first three cases, 16-gauge catheter was used with its needle inserted and the needle withdrawn once the catheter was in position. Catheter removal was sometimes necessary, and a new catheter redirected into the target. Subsequently, a series of progressive scans was devised to slowly advance a titanium needle, while correcting the angle using the sequential scans as the needle was placed in real-time (see text footnote 2). Once, in place, the stylet was withdrawn from the needle, and 1–2 ml of PRP or MSC suspended in PRP was injected. Postinjection transverse STIR FSE sequences were completed in an attempt to document increased fluid signal in the DIJCL.

### Rehabilitation

Postinjection recommendations included absolute stall rest for 1 month with subsequent increased exercise by walking in hand for 2 months and bar shoe application to both front feet. Turnout was not recommended until walking and trotting under saddle was completed without evidence of lameness.

### Outcome Measures

Follow-up was completed by contacting owners by telephone, email, or by examination 1 year or longer after the injection. Information requested at the time of follow-up included whether the horse was sound, and whether the level of exercise was as expected or less than expected (Table 1). A recheck MRI was completed in four horses to evaluate any change in the MRI abnormalities.

**Table 1:** **MRI-guided injection of the DIJCL**.

Horse #	Age (years)	Sex	Breed	Grade of lameness prior to injection	Duration of lameness prior to injection (months)	Type of injection	Level of exercise: expected or less than expected	Time to resolution of lameness (months)	Other MRI lesions	Treatments after injection
1	11	G	Wmbld	2/5	4	Stem cells and PRP	Expected	~8	NB cyst	Unk
2	7	M	Wmbld X	3/5	1	Stem cells and PRP	Expected	12	Associated with DP fracture	Unk
3	10	G	Cleveland Bay	3/5	4	PRP	Expected	8	None	Unk
4	9	G	Selle Fran	NR	3	PRP	Lesser	Unk	Mild NB edema	None
5	8	F	Draft X	3/5	1	Stem cells and PRP	Expected	6	Mild coffin bone edema	Unk
6	17		Swedish Wmbld	NR	6	PRP	Expected	18	None	None
7	5	G	ISH	NR	3	PRP	Expected	7	Navicular bursitis and DIJ synovitis	None
8	14	G	TB X	3/5	3	Stem cells and PRP	Sound not in full work	6	Bilateral navicular bone edema	Hyaluronic acid in the DIJ
9	7	G	TB	2/5	12	PRP	Expected	7	Navicular bursitis	Triamcinolone in the navicular bursa
10	10	G	Dutch Wmbld	3/5	12	Stem cells and PRP	Expected	8	None	None
11	16	G	Crossbred	3/5	8	PRP	Expected	6	None	Unk
12	11	G	TB	2/5	8	Stem cells and PRP	Lesser	6	None	Unk
13	19	G	Irish Wmbld	NR	NR	Stem cells and PRP	Lesser	>18	None	Stem cells in the DIJ and Shockwave

*G, gelding; F, female; M, male; Wmbld, Warmblood; ISH, Irish sport horse; TB, thoroughbred; PRP, platelet-rich plasma; DP, distal phalanx; DIJ, distal interphalangeal joint; MCS, mesenchymal stem cells; NR, not recorded; Ukn, unknown*.

## Results

Signalment, duration and grade of lameness, type of treatment, and outcome are presented in Table 1. All horses were referred, and all had a history of lameness localized to the foot. Lameness resolved with a palmar digital nerve block in three horses, with an abaxial sesamoid block in four horses, up to a low four-point block in one horse and a history of lameness localized to the foot without specifying the nerve block in five horses. One horse had evidence of injury to the collateral ligament on a previous ultrasound examination. Ten horses had no resolution of the lameness after previous treatments, including variable periods of rest, intra-articular treatment of the affected distal interphalangeal joint with triamcinolone, and/or hyaluronic acid (seven), oral phenylbutazone administration (one), and intravenous disodium tiludronate (two). Based on the history of localization of the lameness to the foot and subsequent identification of injury to the DIJCL desmopathy of the DIJCL were considered the primary cause of lameness in 13 horses.

Abnormal MRI signal was found in T1W-GE (9), STIR (7), T2 FSE (12), and PD (11) sequences. Images from one horse were not available to evaluate all sequences. Five horses had DIJCL insertion fossa enlargement, one had cyst formation at the insertion, and one had focal bone inflammation at the insertion. Desmopathy in the horse with hind limb desmopathy was associated with a previous distal phalanx fracture, which affected the collateral ligament at its insertion.

**Figure 2:**
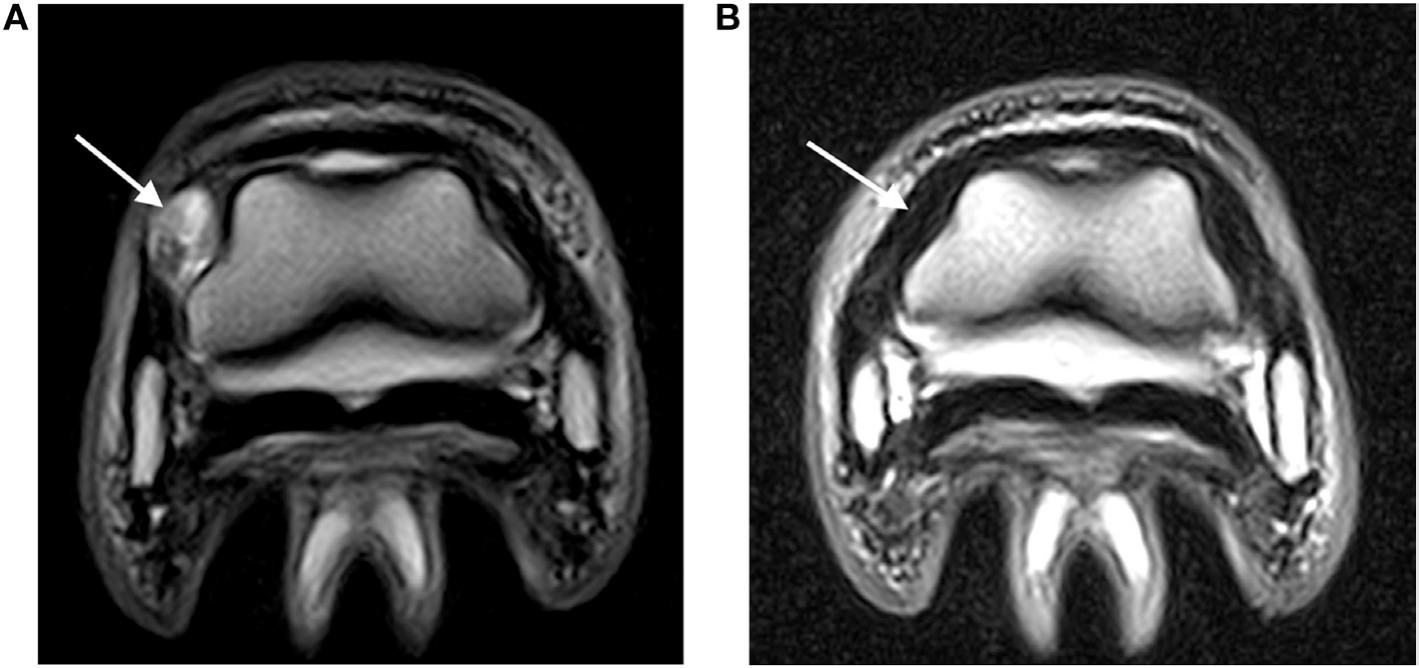
**(A)** Abnormal hyperintense signal (arrow) in a DIJCL prior to injection (T2-weighted fast spin echo sequence; medial is to the left). **(B)** Recheck MRI examination demonstrating normal signal intensity (arrow) in the same DIJCL 6 months after injection (T2-weighted fast spin echo sequence; medial is to the left).

Two horses had two 500 mg doses of flunixin meglumine postinjection, while the remaining horses had no postinjection treatment. No complications after the DIJCL injections were reported. All lameness due to injuries of the DIJCLs resolved. The time for resolution of the lameness ranged from 6 to 18 months (Table 1). Four horses had follow-up MRI examinations, which revealed a partial (two) or complete (two) resolution of the abnormal signal (Figure 2). The presence of improved but still abnormal signal in follow-up MRI examinations of two horses could not be specifically identified as a response to the needle insertion.

Lameness in one horse due to DIJCL desmopathy, navicular bone edema, and navicular bursitis recurred. Repeat MRI 3 months after the initial diagnosis showed marked improvement in the injected collateral ligament, but navicular bone edema and bursitis were unchanged. Lameness resolved with injection of the navicular bursa, but subsequently the horse was retired due to recurring front limb lameness. Lameness in the affected limb in one horse resolved, but the horse was subsequently retired due to rear limb lameness.

## Discussion

Surgical and medical intervention for tendon and ligament injuries has been used for many decades. The goal is to stimulate a healing in an environment, which heals with scar tissue or does not heal due to vascular compromise (24). Lesions found in an injured DIJCL and deep digital flexor tendon consist of disorganized matrix and poor collagen maturation (6, 10, 25). The inability of some of these lesions to resolve may be due to a lack of inflammatory response and/or lack of blood supply with no stimulus for fiber regeneration or remodeling.

Surgical splitting of tendon and ligament lesions is an attempt to release acute core lesion edema and is used to stimulate healing in avascular scar tissue in chronic injuries (24, 26). Because the needle can be accurately placed in the DIJCL using MRI guidance, this disruption of the abnormal tissue may stimulate a cellular response and increased vascularity in the ligament as observed with tendon and ligament splitting without the use of MSC or PRP. Although prolonged stall rest with appropriate shoeing has been successful in resolving lameness in up to 60% of horses with or without additional medical therapy (9), all horses in our study had prior rest, which was not successful in resolving the lameness prior to injection.

Needle placement in the DIJCL is improved using MRI guidance compared with use of radiographs or ultrasound (21). The needle position is identified as focal decreased signal intensity in the tissue (Figure 1). The technique has been improved by use of overlapping two slice T1W-GE sequences, which can be completed in real time sequentially as the needle is advanced (see text footnote 2). This allows visualization of and redirection of the needle placement as it is being advanced into the ligament. Additionally, this decreases the time required for the injection and helps complete needle placement with minimal redirection. Use of navigational ultrasound imaging, which can correlate previous MRI images with ultrasound for real-time guidance during interventional therapies, may provide an additional method for injection of the DIJCL (27). This appears to be an advantage for lesions identified by high-field MRI that can subsequently be treated without general anesthesia. However, it is not clear if this technique will allow accurate injection in the distal DIJCL, where ultrasound cannot penetrate the hoof.

Response to rest and corrective shoeing for DIJCL injuries is reported at 5 of 17 horses (29%) returning to full work with an additional 2 horses improved for light work and breeding (8). Rest, medical treatment, and supportive shoeing resulted in 60% resolution of lameness in horses with DIJCL desmopathy (9). Horses with DIJCL desmopathy commonly have concurrent changes in the navicular bone, deep digital flexor tendon, and impar desmopathy making it difficult to determine the source of pain (13, 28). Horses with deep digital flexor lesions detected by MRI have a worse prognosis that horses with other types of injury, including DIJCL desmopathy. Seven of the 13 horses in this study had concurrent lesions in the navicular bone (6) and distal phalanx (1). None of the horses in this group had deep digital flexor tendon lesions likely improving the chance of successful treatment (13). Although concurrent lesions in seven horses could have contributed to the lameness, the DIJCL changes seen on MRI were considered the primary problem, which required treatment based on the history and character of the lesions.

This study confirms the ability to accurately inject the DIJCL in standing horses using sedation and local anesthesia for low-field MRI. Although the treatment appears beneficial, this study did not confirm efficacy, as there is no direct comparison for cases treated with rest, as previously reported (13, 28). Furthermore, there was no control for the potential stimulation created by needle placement alone.

## Conclusion

Magnetic resonance imaging-guided injection of stem cells or PRP into the DIJCL is a safe and repeatable technique in standing horses. Further studies of regenerative medical therapy for the DIJCL are needed to confirm the benefit of this therapy.

## Author Contributions

Both authors, NW and JB, were involved with all aspects of the study, including diagnosis, treatment, data collection, review of the data, and authoring the manuscript.

## Conflict of Interest Statement

Dr. JB is on the scientific advisory board of ReCellerate, Inc. The remaining author declares that the research was conducted in the absence of any commercial or financial relationships that could be construed as a potential conflict of interest.

## Footnotes

^1^IRAP II, Arthrex Vet Systems, Naples, FL, USA

^2^Hallmarq Veterinary Imaging Inc., Acton, MA, USA

^3^Puncture needle, *Invivo*, Gainesville, FL, USA

## Abbreviations

DIJCL, distal interphalangeal joint collateral ligament; MRI, magnetic resonance imaging; MSC, mesenchymal stem cells; PRP, platelet-rich plasma; STIR FSE, short-tau inversion-recovery fast spin echo sequence; T1W-GE, T1-weighed gradient echo sequence.
